# Prosecuting cases of abusive head trauma in Switzerland: a descriptive study of the impact of medical documentation and delay of reporting on judicial outcome

**DOI:** 10.1007/s00414-024-03212-4

**Published:** 2024-03-28

**Authors:** Sarah Held, Jean-Jacques Cheseaux, Jean-François Tolsa, Sarah Depallens

**Affiliations:** 1 Faculté de Biologie Et de Médecine, UNIL, Lausanne, Switzerland; 2 Service de Pédiatrie, CHUV, Lausanne, Switzerland

**Keywords:** Abusive head trauma (AHT), Shaken baby syndrome (SBS), Subdural hemorrhages (SDH), Retinal hemorrhages (RH), Childhood protection service, Child abuse

## Abstract

Abusive head trauma (AHT) is a criminal offence that is prosecuted ex officio, following report to the police from physicians or child protection services. The aim of this study was to assess whether the judicial outcome (dismissal vs indictment) was influenced by the quality of the medical documentation and/or the time span between AHT diagnosis and reporting child abuse to the police. The cohort was divided in two groups: 13/23 dismissals (57%) and 10/23 indictments (43%). The diagnostic probability of the AHT cases was certain for both groups. Nonetheless, in fraction of dismissed cases, alternative explanations for the observed lesions seemed plausible to the public prosecutor. Legal files of only 3/12 dismissed cases had a forensic report, while 6/10 cases that were indicted included a forensic report. Further, the legal file of several dismissed cases entirely lacked medical documentation (3/12), which was not the cases for indicted cases. The period between AHT diagnosis and reporting to the police was not different for dismissals (29 ± 19 days) and indictments (7 ± 4 days) (*p* = 0.32). Physicians filed reports more rapidly (6 ± 1 days) compared to childhood protection service (70 ± 46 days) (*p* = 0.01) and that may increase the rate of indictments (9/18) compared to reporting via the childhood protection service (1/5). Despite diagnostic certainty, other causes for the lesions were considered as plausible alternative explanations to judicial professionals in several dismissed cases. These seemed to have less medical documentation and forensic evaluations. In addition, more rapid reporting to the police by physicians seems to increase the likelihood of indictments.

**Supplementary Information:**

The online version contains supplementary material available at 10.1007/s00414-024-03212-4.

## Introduction

Abusive head trauma (AHT), previously called shaken baby syndrome (SBS), is a serious brain injury resulting from forcefully shaking an infant [1–3]. The incidence of AHT in Switzerland is 14/100′000 live births, with a majority of boys affected by this form of child abuse [4]. The most frequently affected age group is under 6 month [2]. This repetitive form of abuse [7] may lead to irreversible neurological damage and death [3–5].

The main criteria to diagnose AHT, that were established by the “Comity of High authority of health in France” (HAS) [2] includes: Intracranial injuries, including subdural hematomas (SDH) [4, 6, 7], spinal cord injuries [8] and retinal haemorrhages (RH) [4, 9]. Additional lesions such as fractures and skin lesions are present in 80–85% of cases [4, 10]. These can be related to the process of shaking or more general child abuse [11, 12]. Non-specific neurological symptoms (vomiting, apnoea, seizures etc.) are present in 85% of cases [4]. The probability of the AHT diagnosis depends on the association of the intracranial injury (SDH, RH) and associated clinical features (fractures, skin lesions, neurological symptoms) [12]. The main differential diagnosis is accidental head trauma due to a high-energy traumatic event, which must be excluded [2].

Once diagnosed, the suspicion of child abuse can be reported to the police directly by health care professionals (after lifting the medical secret), or indirectly by the childhood’s protection service following a notification by a physician (no lifting of medical secret necessary). AHT is a criminal offence that is prosecuted ex officio. The public prosecutor eventually decides to dismiss the case (i.e. if there is insufficient evidence for abuse or if a culprit cannot be identified). Alternatively, he will indict a suspect and thus open court proceedings. A criminal court ultimately decides to convict or acquit a suspect of the criminal charges.

The aim of this study was to identify factors that influence the rate of indictments in the case of AHT. Specifically, we assessed whether the judicial outcome to dismiss a case or indict a suspect was influenced by the quality and type of the medical documentation and/or the time span between diagnosis and reporting of the suspicion of child abuse to the police. As convicted perpetrators can receive psychological help, and victims can receive better long-term medical and social support, the long-term goal is to improve child protection.

## Method

### Study design and inclusion criteria

For this retrospective qualitative study, we analysed cases of AHT admitted to the University Hospital of the Canton de Vaud (CHUV) between 2001 and 2016.

Patients for the study were identified based on the records from the Child Abuse and Neglect Team (CAN Team). Infants < 2 years were eligible for inclusion if they had undisputed diagnosis of AHT based on medical documentation. AHT diagnosis relied on cerebral imagery, fundoscopy and anamnestic/clinical findings as described in the recommendations of the HAS [2]. Medical data were collected from the internal archiving system of the CHUV and the data bank of the CAN team.

In addition, cases were included when the public prosecutor or courts granted access to legal file and when the judicial case was closed. Judicial data were collected by the prosecutor’s offices and courts of the canton in which the judicial files had been completed. The judicial files were evaluated regarding the.

- who filed the complaint (health care professionals or childhood protection service)
- the timespan between hospital admission and filing of report to the police
- presence of medical documentation: discriminating between absence of medical documentation (none); presence of one type of documentation (single); presence of more than one documentation (multiple)
- type of medical documentation: discriminating between a forensic report issued by the University Center of Legal Medicine (CURML), a report from the CAN Team and/or the presence of clinical files (i.e. paediatric hospitalisation letters or specialist’s reports)
- motive for shaking
- plausible alternative explanations for the presence of lesions according to the court
- justification for specific legal outcomes (dismissals versus indictments, convictions versus acquittals)

Patients were divided into two groups, based on the public prosecutors’ decision to either dismiss the case or indict a suspect.

### Outcomes

We first addressed the hypothesis that the quality and type of the medical documentation included in the judicial file impacted the outcome (dismissal or indictment).

We then assessed whether the timespan between the admission to the hospital and the reporting of the suspected child abuse to the police affected the judicial outcome (dismissal or indictment).

### Ethical considerations

This study was approved by the Ethical Committee CER-VD (n° 2017–00436) for the use of medical data without consent.

The public prosecutor of each canton decided whether the legal representatives had to give their authorisation to access the judicial data.

### Statistical analysis

**Fig. 1:**
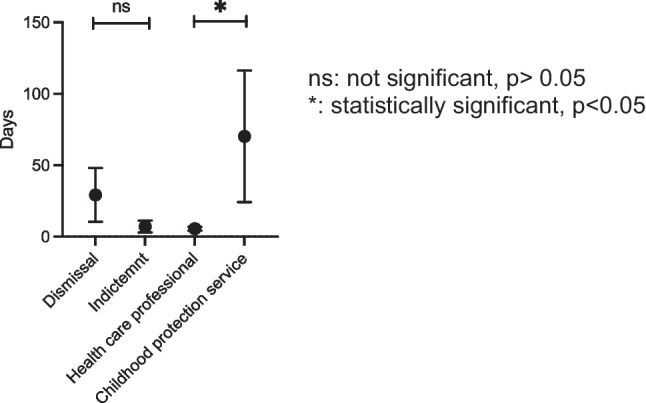
Time span between diagnosis and filing a report depending on judicial outcome or author filing the report. ns: not significant, *p* > 0.05. *: statistically significant, *p* < 0.05

The dot graph (Fig. 1) shows mean ± SEM [days]. The statistical pairwise analysis of durations [days] is based on students t-test. For statistical comparison of the number and type of medical documentation in legal files and author filing charges, a chi-squared test was used. Statistically significance is indicated as * when p < 0.05, ** when p < 0.01, and not significant (ns) when *p* > 0.05.

### Legal definitions (Switzerland)

Abusive head trauma (AHT) is a criminal offence that is prosecuted ex officio, following reporting from relevant institutions. In the Canton de Vaud, the report can be filed directly by health care professionals or indirectly by childhood protection services following the notification by health care professionals. For health care professionals, medical secret has to be lifted by the cantonal doctor. Reports will go to the police, who will make the first investigation. The case moves ex officio to public prosecution, the public prosecutor eventually decides to dismiss a case (e.g. due to insufficient evidence against a suspect or inability to identify a suspect) or to press charges (indict) a suspect, which leads to court proceedings. A court/judge eventually decides to acquit a suspect of the charges or to convict.

Limitation period: is a legal period during which the justice system has to act. Once this period has passed, it is no longer possible to prosecute a suspect.

## Results

### Patient distribution and AHT diagnosis

This study initially included 33 patients with AHT diagnosis. A total of 10 cases had to be excluded since the legal files were not accessible (*n* = 5), the legal procedure was still pending (*n* = 4) or the parents rejected the access to file (*n* = 1). A total of 23 cases were enrolled in this study. The cohort was divided into two groups according to the judicial outcome: indictment (10/23) or dismissal (13/23).

The characteristics and the diagnosis of the patients in the indictment (10/23) versus the dismissal (13/23) groups are detailed in Table 1.

**Table 1:** Distribution and AHT diagnosis of patients

Characteristics		Indictments *n* = 10/%	Dismissals *n* = 13/%
Sex	Female	4(40)	8(61.5)
	Male	6(60)	5(38.5)
Age [month]	Mean	5,3 ± 3,1	5,2 ± 2,1
	Median	4,5	5
Deceased		1(10)	1(7.7)
Diagnostic criteria			
Subdural hematoma	Unique	2(20)	0
	Multiple	8(80)	13(100)
Other intracranial lesions	None	1(10)	2(15.4)
	ICH/SAH	6(60)	7(53.8)
	Hypoxic-anoxic	4(40)	4(30.8)
	ICHT	5(50)	4(0.8)
	Venous throbosis	0	1(7.7)
Retinal hemorrages	None/no exam	1(10)	3(23)
	Unilateral	1(10)	2(15.4)
	Bilateral	8(80)	8(61.5)
Associated critera			
Fractures	Skull (multiple)	0	1(7.7)
	Skull (unique)	3(30)	1(7.7)
	Vertebral (cervical)	0	1(7.7)
	Long bones and ribs (multiple)	4(40)	1(7.7)
	Long bones and ribs (unique)	0	1(7.7)
Skin lesions	Ecchymosis, hematomas (multiple)	6(60)	3(23)
	Ecchymosis, hematomas (unique)	0	2(15.4)
	Burns	1(10)	0
Differential diagnosis			
Accidental head trauma (1)		0	0
Coagulopathy	Minor anomaly (2)	4(40)	1(7.7)
	Negative	6(60)	11(84.6)
Menkes Disease	Tested negative (3)	5(50)	8(61.5)
	Not tested	5(50)	5(38.5)
Type 1 glutarc aciduria	Tested negative	10(100)	12(92.3)
	Not tested	0	1(7.7)
Cerebral arteriovenous malformation		0	0
Diagnostic probability *HAS			
	Certain	10(100)	13(100)
	Propable	0	0

(1) We defined accidental head trauma as negative anamnesis of high energy trauma and objectivized clinical lesion suggestive for AHT

(2) Cannot explain spontaneous SDH and/or RH

(3) Tested: 8/11 boys and 5/12 girls

### Timespan between diagnosis and reporting suspicion of AHT

The time span between AHT diagnosis and the filing of a police report for dismissed cases (29 ± 19 days, mean ± SEM) was not different from cases that led to indictments (7 ± 4 days) (*p* = 0.32; student’s t-test) (Fig. 1).

Once AHT is diagnosed, the police is notified directly by health care professionals or indirectly by the childhood’s protection service following a notification from a physician. After a first investigation, the cases move ex officio to public prosecution. When a report was filed by physicians, the time between diagnosis and reporting was 6 ± 1 days. The time span was considerably longer (70 ± 46 days) when the report was filed by the childhood protection service (*p* = 0.01; student’s t-test) (Fig. 1).

**Fig. 2:**
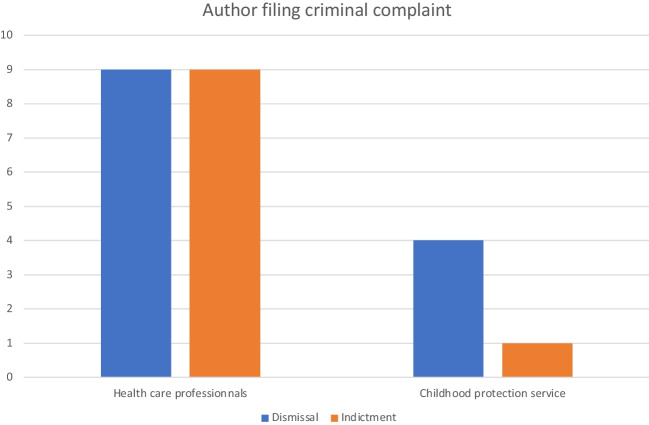
Author filing criminal complaint

Health care professionals filed reports directly in 18/23 (78%) cases, while reports were filed by the childhood protection service in 5/23 (22%) cases. In the first group, 9/18 cases were dismissed and 9 led to an indictment (50%) (Fig. 2). In the second group, 4/5 cases were dismissed and only 1 resulted in an indictment (20%) (*p* = 0.23; chi squared test) (Fig. 2). Even though these data are not statistically significant, they nevertheless raise the possibility that an accelerated reporting increases the rate of cases that result in indictments.

### Relation of medical documentation to judicial outcome

Judicial files were analysed for number and types of medical.

**Fig. 3:**
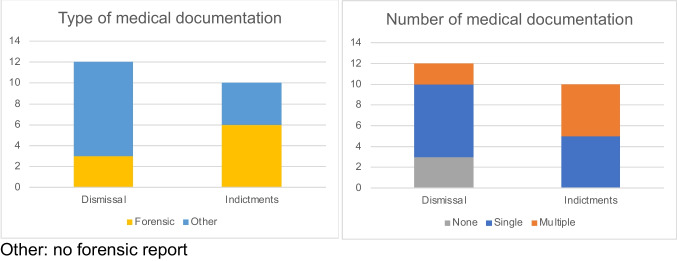
Type and number of medical documentations in judicial files. Other: no forensic report

One of 13 patients in the dismissal group had to be excluded as the judicial file had been transferred to a foreign country during data collection. The charges had been dismissed in Switzerland. In the remaining 12 dismissed cases only 3/12 had a forensic report, while 6/10 cases in the indictment group included forensic reports (*p* = 0.096, chi-squared test) (Fig. 3).

Further, 3 of the 12 dismissed cases had no additional medical documentation, while all cases in the indictment group included at least one additional medical documentation (Fig. 3). Conversely, 5/10 cases in the indictment group had multiple documentations, which was only the case for 2/12 dismissed cases (*p* = 0.11, chi squared test) (Fig. 3). There is thus a trend to better medical documentation in the indictment group. For more details see Table S1 (Appendix).

### Admission of shaking and judicial hypothesis for medical lesions and justification for judicial outcomes

#### Dismissal

In 4 of the 13 cases that were dismissed shaking was admitted. In these cases, shaking was explained by an attempted reanimation following a sudden loss of consciousness of the infant (4/13). Legally the dismissals were justified by insufficient evidence of intentional shaking or neglect (2/13) and exceeded limitation period (legal period during which the justice system must act, once passed, it is no longer possible to prosecute a suspect) (2/13).

Shaking was not admitted in 9/13 dismissed cases. Despite the fact that AHT diagnosis was certain to the physician, alternative explanations for the lesions seemed plausible to the public prosecutor. These explanations included accidental head trauma 30.8% (4/13), a game 15.4% (2/13), birth trauma resulting from the use of forceps and/or the side effects of vaccines (1/13). Legal files did not include any explanation for the symptoms in 2/13 cases.

The legal justification for dismissals were: the inability to prove the identity of the offender (7/13) or insufficient proof of guilt (2/13).

#### Court Proceedings

In all cases that resulted in court proceedings (10/10), shaking was admitted.

Nine of the court proceedings resulted in convictions. In these cases, children were shaken based on crying (6/9), justified by an attempt to reanimate a child (1/9) or no obvious reason (2/9). Legally, perpetrators were convicted for negligent homicide (1/9) or simple body injury, grave bodily harm (by neglect), assault, violation of the duty to assist or educate and/or exposure offences (8/9).

One of the court proceedings ended in an acquittal (1/10). Here, the child was known to suffer from apnoea due to gastroesophageal reflux and resuscitations measures by shaking seamed plausible to the court in the context of the stressful situation.

In summary, during the judicial process shaking was admitted in 65% of all cases (14/23), 10 of which resulted in court proceedings. In all 10 cases, the court proceedings were the consequence of confessions. For more details see Appendix (Figure S1).

### Limitations and strength of the study

The most limiting factor of this study is the small number of cases, which is partly due to the small number of suspected AHT cases. In addition, AHT diagnosis may be uncertain, and we deliberately decided to only include cases with certain diagnosis in order to assess the fate of certain AHT diagnoses in court proceedings. These limitations reduced the number of victims to *n* = 23 that were treated at the CHUV over a period of 15 years.

This study, unique in Switzerland, made it possible for the first time to compare medical records with judicial records. This type of research is key to improve the collaboration between medical and legal communities to improve childhood protection by identifying the abuse and thus preventing possible recurrences. This study should be expanded to other centers to confirm and extend of our results.

## Discussion and conclusions

The diagnosis of AHT which was based on the diagnostic criteria of the HAS [2] was similar for the dismissal and the indictment groups. The differential diagnosis of AHT, such as accidental head trauma, bleeding disorders and metabolic disease were ruled out in all of our cases. The diagnostic probability of AHT was certain for both groups. It was thus surprising to note that 57% of the cases were dismissed, in a similar study investigating criminal proceedings in AHT in Germany, 50% of the proceedings were closed [13]. Furthermore, public prosecutors relatively frequently considered alternative explanations as plausible causes for the medical lesions, which included accidental head trauma, a game, birth trauma resulting from the use of forceps and/or the side effects of vaccines. However, according to the medical literature, accidental head trauma presents rarely with SDH, and hardly ever if SDH is associated with RH and fracture [14]. The HAS also points out that traditional games (for example with a baby bouncer chair) never induce lesions found in AHT patients [2] and that reanimation in infants does not result in costal fractures [15, 16], SDH or RH [2, 17–19]. Notwithstanding, many of these cases were dismissed or acquitted. Therefore, even though the diagnostic probability of AHT was certain, it appeared very difficult to indict a suspect if he/she did not admit shaking.

We further noted that, the type and number of the medical documentation present in the judicial files was variable. Overall, cases that were dismissed had less forensic reports and more frequently lacked additional medical documentation compared to cases that led to court proceedings.

In addition, a rapid filing of reports to the police seemed to increase the likelihood of indictments. This timespan was clearly shorter when healthcare professionals filed reports directly compared the indirect report via the childhood protection services. A possible explanation for this observation is that witnesses remember recent events more accurately and that there is less time for relatives to protect the offender or to arrange their defence.

Irrespective of the medical factors discussed above, it is important to keep in mind, that additional factors play important roles in the decision to dismiss a case. For example, the non-identification of the culprit or insufficient evidence of guilt were the predominant reasons for dismissal. These factors are also described in the study of Feld et al. [13].

The central aim of identifying and prosecuting culprits is to prevent further shaking or other forms of child abuse. On the one hand, prosecution provides culprits with the opportunity to recognize child abuse and obtain psychiatric support to change the violent behavior. On the other hand, the recognition as a victim, gives the child the possibility to obtain better medical and social long-term support and protection.

These observations show that a diagnosis that is certain to medical professionals are not necessarily sufficiently certain to stand before justice, therefore we suggest:

- Thorough medical documentation including all diagnostic criteria and exclude differential diagnosis with a clear statement of position regarding the diagnosis.
- Forensic evaluation for all cases, that can transmit the findings in a more accessible fashion to non-healthcare professionals.
- Multiple types of medical documentation that reach the same conclusion/diagnosis of AHT, may reduce the plausibility of alternative explanations for the lesions.
- Direct and rapid filling of a report to the responsible judicial once diagnosis is set.

### Supplementary Information

Below is the link to the electronic supplementary material.

Supplementary file1 (DOCX 73 KB)

## Data Availability

Because of the high sensitivity of the judicial data no public data will be shared. On request, access could be discussed.
