# Supplementary material for: Prosecuting cases of abusive head trauma in Switzerland: a descriptive study of the impact of medical documentation and delay of reporting on judicial outcome
Source: Int J Legal Med. 2024 Mar 28;138(4):1645–51. doi: 10.1007/s00414-024-03212-4 (PMC11164792; doi:10.1007/s00414-024-03212-4)
Supplement: Supplementary file 1 — Supplementary file1 (DOCX 73 KB) [file 414_2024_3212_MOESM1_ESM.docx]

# Appendix

**Figure S1: Admission of shaking and judicial hypothesis for medical lesions and justification for judicial outcomes**

**§**

**Table S1 : Distribution of medical documentation in legal file**
